# Evaluation of potential risk of transmission of avian influenza A viruses at live bird markets in response to unusual crow die‐offs in Bangladesh

**DOI:** 10.1111/irv.12716

**Published:** 2020-01-07

**Authors:** Mahbubur Rahman, Punam Mangtani, Timothy M. Uyeki, Jacqueline M. Cardwell, Montserrat Torremorell, Ariful Islam, Mohammed A. Samad, A. K. M. Muraduzzaman, Md Giasuddin, Sudipta Sarkar, A. S. M. Alamgir, M. Salimuzzaman, Meerjady S. Flora

**Affiliations:** ^1^ Royal Veterinary College Hatfield UK; ^2^ Institute of Epidemiology Disease Control and Research Dhaka Bangladesh; ^3^ London School of Hygiene and Tropical Medicine London UK; ^4^ Influenza Division Centers for Disease Control and Prevention Atlanta GA USA; ^5^ University of Minnesota Twin Cities MN USA; ^6^ EcoHealth Alliance New York NY USA; ^7^ Bangladesh Livestock Research Institute (BLRI) Savar Bangladesh

**Keywords:** avian influenza, avian influenza A virus, Bangladesh, influenza in birds, live bird market, pathogen transmission

## Abstract

In response to unusual crow die‐offs from avian influenza A(H5N1) virus infection during January‐February 2017 in Dhaka, Bangladesh, a One Health team assessed potential infection risks in live bird markets (LBMs). Evidence of aerosolized avian influenza A viruses was detected in LBMs and in the respiratory tracts of market workers, indicating exposure and potential for infection. This study highlighted the importance of surveillance platforms with a coordinated One Health strategy to investigate and mitigate zoonotic risk.

## 1 INTRODUCTION

Bangladesh has reported 556 outbreaks of highly pathogenic avian influenza (HPAI) A(H5N1) virus in poultry and wild birds since 2007.^1^ Sporadic human infections with avian influenza A viruses (AIVs), including eight patients with A(H5N1) virus infection and one fatal outcome, were reported.^2^ A study conducted between May 2012 and December 2015 across Bangladesh reported high sero‐prevalence of A(H5N1) virus antibodies among house crows (*Corvus splendens*) feeding on offal from live bird markets (LBMs).^3^ In 2011 and 2016, A(H5N1) virus spread among house crows in several districts of Bangladesh, including the capital, Dhaka.^4^, ^5^ Crow mortality from HPAI A(H5N1) viruses was also reported in Japan and India.^6^, ^7^ Crow deaths were investigated previously in Dhaka,^4^ whereas this investigation assessed possible sources of HPAI A(H5N1) virus and potential spillover risks to humans.

## 2 INITIATION OF OUTBREAK INVESTIGATION

In response to a report of unusual crow mortality around central Dhaka, on January 14, 2017,^8^ the Institute of Epidemiology, Disease Control and Research (IEDCR) initiated a multidisciplinary investigation from January 21 to February 12, 2017. The wildlife team identified 124 crow deaths within 7 km of the initial reported crow roosts.^8^ Crow samples tested positive for A(H5N1) virus by real‐time reverse transcription PCR (rRT‐PCR).^8^ The hypothesis that crows might have acquired A(H5N1) virus infection after consuming infected dead poultry and/or their waste products^3^ was based on the observed crow die‐offs near LBMs, where crows were observed feeding on poultry offal. To assess occupational risk of infection to exposed humans, we conducted a cross‐sectional survey, examined air samples in LBMs, and collected respiratory samples from workers for influenza testing at 10 LBMs near the crow die‐offs.

## 3 METHODS

For markets with ≤20 workers, we recruited all employees; while in markets with >20 workers, we randomly selected 20 workers per market. We collected nasal and throat swabs, and information on illness symptoms and workers’ practices using a semi‐structured questionnaire. In each market, air samples were also collected by a liquid cyclonic air sampler,^9^ together with market‐level hygiene assessments through observation. Based on a previous study, one air sampler was used for 30 minutes, placed in the center of each LBM, 1.3 meters from the ground and approximately 0.5 m from poultry housing^10^ during 10:30 to 11:30 AM. At each LBM, the animal health team collected swabs from fresh fecal droppings beneath the poultry cages and accumulated offal samples; four samples from random sites were pooled together as one environmental sample per LBM. All samples were tested using rRT‐PCR with appropriate positive and negative controls to exclude contamination. Nasal and throat swabs were tested for influenza A and B viruses with subtyping of M‐gene‐positive influenza A viruses for seasonal H3, H1N1pdm09, and avian H5/H7/H9.^11^ Influenza A positive air and pooled environmental samples were subtyped for avian H5/H7/H9 only. Descriptive analysis was conducted using statistical software STATA (version 14.2).

## 4 RESULTS AND DISCUSSION

Of the 10 markets, three had <8 poultry stalls, and the rest had 9‐16 stalls. The average number of workers per stall was 3.0 (SD 1.1). Almost all stalls slaughtered and defeathered birds. Multiple species of poultry were sold, including chickens, pigeon, geese, quail, and ducks at seven markets, and three LBMs only sold chickens. In all LBMs, birds not sold at the end of the day were kept in the same stall. No LBMs practiced market closure days or rest days without poultry.

All LBMs had visible poultry feces on the ground, but no dead birds. Six LBMs reported market cleaning more than once daily, but only one used disinfectant (eg, bleach). Three LBMs reported disposal of solid waste at least twice daily. Half of the markets had open drains. About 60% of stalls experienced poultry deaths in the week before the investigations, and some workers reported discarding poultry carcasses as garbage or giving them to other workers. Temperature and relative humidity were not significantly different among LBMs during air sampling.

We enrolled 151 workers from 81 stalls, with mean age of 31.3 (SD 11.8) years and median work experience of 9.0 years, interquartile range (IQR) 4‐16 years, and all except one were male. Nearly 40% of the workers reported one or more of the following signs and symptoms in the previous 10 days: fever/feverishness (11.3%); measured temperature of ≥100.4°F (2.0%); cough (15.2%); sore throat (6.0%); runny nose (23.8%); eye redness (2.0%); diarrhea (0.7%); difficulty breathing (4.6%); headache or body ache (11.3%); and 60.9% were asymptomatic. Three workers reported febrile respiratory symptoms, and all three tested negative for influenza viruses by rRT‐PCR. Overall, 21 (13.9%) LBM workers had respiratory specimens that tested positive for influenza A (12.6% of nasal swabs, 4% of throat swabs), of whom 62% were asymptomatic and only four (19%) reported respiratory symptoms (runny nose and/or cough) without fever. Six LBMs had at least one worker who tested positive for influenza A virus. Most of the influenza A positive samples were either H9 or non‐subtypeable (Table 1).

**Table 1:** Comparison of rRT‐PCR results of influenza A viral RNA detection for live bird market (LBM) air samples and workers’ respiratory (nasal and throat) specimens, Bangladesh, 2017^a^

Air sample result (n = number of LBMs tested)^b^	No. of LBM workers tested	No of LBM workers with influenza A positive specimens^c^	% (95% CI) of LBM workers with influenza A positive specimens	Influenza A virus subtype detected from human respiratory specimens				
				H5 n	H7 n	H9 n	Both H5 & H9 n	Non‐subtypeable n
Influenza A(H5) and A(H9) (5)	71	12	16.9 (9.0‐27.7)	1	0	5	1	5
Influenza A(H9) (4)	59	3	5.1 (1.1‐14.2)	0	0	2	0	1
Negative (1)	21	6	28.6 (11.3‐52.2)	0	0	3	1	2
Total (10)	151	21	13.9 (8.8‐20.5)	1	0	10	2	8

^a^ rRT‐PCR, real‐time reverse transcription PCR; LBM, live bird market.

^b^ rRT‐PCR of samples was conducted in International Centre for Diarrhoeal Disease Research, Bangladesh (icddr,b), Dhaka, Bangladesh; Culture of 3 M‐gene‐positive air samples was conducted at the Bangladesh Livestock Research Institute (BLRI), Savar, Bangladesh.

^c^ rRT‐PCR of human respiratory samples was conducted at the Institute of Epidemiology, Disease Control and Research, Dhaka, Bangladesh; The cutoff for Influenza A positivity in rRT‐PCR assays was a cycle threshold (*C*_t_) of ≤ 38; None of the nasal or throat swab specimens tested positive for seasonal influenza A(H1N1)pdm09, A(H3), or Influenza B viruses.

All 10 LBMs had pooled environmental specimens that tested positive for influenza A, including five H5, one with both H5 and H9, and four markets with non‐subtypeable specimens. AIVs were detected by air sampling at nine of 10 LBMs, including four with H9, and five with co‐detections of both H5 and H9 (Table 2). Culture of three M‐gene‐positive air samples in embryonated chicken eggs yielded viable virus isolates from three different LBMs, including one A(H5N1) and two A(H9N2) viruses. Sequencing of the HA gene of H5 positive air samples revealed a clade 2.3.2.1a virus that circulated since 2011^4^ and was similar to the partial sequencing results from crow samples in the current outbreak.^5^

**Table 2:** Comparison between rRT‐PCR results for influenza A viral RNA in environmental pooled samples and air samples at 10 LBMs, Bangladesh, 2017^a^

Environmental sample^b^	Air sample^b^				
	Negative	Influenza A(H5)	Influenza A(H9)	Influenza A(H5) & A(H9)	Total
Influenza A(H5)	1	0	2	2	5
Influenza A(H5) and A(H9)	0	0	0	1	1
Influenza A/non‐subtypeable	0	0	2	2	4
Total	1	0	4	5	10

^a^ rRT‐PCR, real‐time reverse transcription PCR; LBM, live bird market.

^b^ rRT‐PCR of environmental and air samples were conducted in International Centre for Diarrhoeal Disease Research, Bangladesh (icddr,b), Dhaka, Bangladesh; The cutoff for Influenza A positivity in rRT‐PCR assays was a cycle threshold (*C*_t_) of ≤ 38.

Our study had several limitations. First, we collected air and respiratory samples only from the identified 10 LBMs in proximity to the seven km area of the crow die‐offs. Therefore, we could not ascertain whether detection of influenza A viruses was limited to these 10 LBMs or whether the high prevalence was because of the overall persistence and amplification of AIVs in LBMs and seasonality. Second, we were unable to confirm the sources of the viruses, whether crows were infected by contact with poultry at LBMs or whether they were responsible for spread of viruses among poultry or between LBMs, or other sources. However, Zhou et al isolated AIVs at LBMs in China^10^ and Bertran et al have demonstrated that experimental processing of HPAI A(H5N1) virus‐infected chickens and ducks generated infectious droplets and aerosols.^12^, ^13^ Overall, LBMs were reported to be highly contaminated with AIVs by multiple studies in Bangladesh and other countries.^14^, ^15^ Taken together, these studies suggest that the most likely source of the aerosolized AIVs in LBMs in Bangladesh is from slaughtering of infected poultry. The crows as carrion eaters were likely infected from the offal or wastage of infected poultry. Third, we did not evaluate workers’ practices for the occupational risk assessment because they had similar work practices as all participated in slaughtering and defeathering. Fourth, several influenza A positive respiratory samples were non‐subtypeable by RT‐PCR. We hypothesized that (a) viral load was too low as most of the non‐subtypeable specimens were collected from non‐ill workers that yielded high Ct values, or (b) the virus subtype was different from those that we tested for. Fifth, detection of AIV RNA in respiratory specimens of healthy LBM workers cannot distinguish asymptomatic infection from contamination. We believe it is more likely that such detection represents the latter, but additional studies are needed. For example, testing to detect viral replication in serial respiratory specimens and serological testing of paired sera to assess seroconversion might provide evidence for infection. Sixth, viral culture was performed on three of nine M‐gene‐positive air samples, and although all three yielded AIVs, it is unknown if viral RNA detection in all of the positive air samples represented exposure to viable aerosolized viruses. Furthermore, since only partial sequencing of influenza A(H5) viral hemagglutinin was performed on H5 positive samples collected from air samples, we could only conclude that the clade 2.3.2.1a H5 viruses were similar to A(H5) viruses detected in crows but might not have been identical to clearly link the crows to infected poultry in LBMs.

## 5 CONCLUSIONS

Overall, we found evidence of aerosolized AIVs in LBMs and respiratory specimens of LBM workers while investigating potential links between crow die‐offs from HPAI A(H5N1) virus and poultry sold at LBMs. House crow die‐offs appeared to be an indicator of the presence of HPAI A(H5N1) viruses in poultry at LBMs and triggered the need for a multidisciplinary investigation to prevent or detect a potential AIV outbreak and assess the risk of exposure and spread to workers. These investigations also highlighted the importance of surveillance platforms (air, environmental, poultry, and human sampling for influenza testing) with a coordinated One Health strategy to investigate zoonotic risk. While detection of AIV RNA in respiratory specimens of workers, especially asymptomatic persons, does not confirm infection, it provides evidence of exposure to AIVs and suggests potential for human infection. These findings indicate that improvements in market hygiene and biosecurity are needed in LBMs to reduce workers’ exposures to AIVs.

## CONSENT TO PARTICIPATE

All the participants provided informed written consent prior to their inclusion in the study.
